# Supplementary material for: Nutritionally recommended food for semi- to strict vegetarian diets based on large-scale nutrient composition data
Source: Sci Rep. 2018 Mar 12;8:4344. doi: 10.1038/s41598-018-22691-1 (PMC5847509; doi:10.1038/s41598-018-22691-1)
Supplement: Supplementary file 1 — Supplementary Information [file 41598_2018_22691_MOESM1_ESM.pdf]

Nutritionally recommended food for semi- to strict vegetarian diets based on large-scale nutrient composition data

Seunghyeon Kim, Michael F. Fenech & Pan-Jun Kim

Supplementary Information

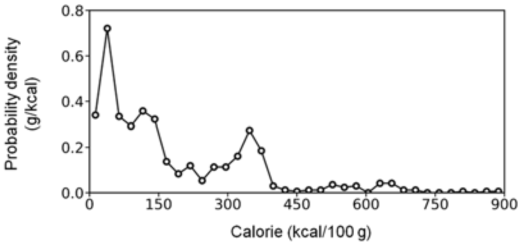

Supplementary Figure S1. Distribution of food calories. Plotted is the distribution of the probability *P*(*x*) that a given raw food has a calorie density of *x*. *x* is measured in kcal per 100 g food; as listed in Supplementary Data S1. The calorie density of each food follows a bimodal distribution with an antimode at ~200 kcal/100 g. Accordingly, we classify foods with ≤ 200 kcal/100 g as low-calorie foods in this study.

Supplementary Table S1. Daily recommended nutrient intakes adapted for irreducible food sets. The first column lists the IDs of the nutrients in the Dietary Reference Intakes (DRI). For the calculation of daily recommended energy (calorie), refer to Supplementary Methods. When obtaining irreducible food sets, we do not impose any lower bound of sodium intake under the assumption that the recommended sodium intake is readily achievable through the consumption of added salt, not necessarily only through raw food consumption. Diets (superscripts, the details provided in Supplementary Methods): C, control diet; O, ovo-lacto vegetarian diet; V, vegan diet; M, methionine-restricted diet; **I**, personalised diet **I** (61-year-old male); **II**, personalised diet **II** (58-year-old female). Other acronyms and symbols: ND, not determined; NA, not applicable (in the case of a vegan diet, we do not set any lower bound of vitamin B_{12} intake for the technical reason described in Supplementary Methods); RAE, retinol activity equivalents; NE, niacin equivalents; DFE, dietary folate equivalents; g/kg, gram per kg body weight; *This upper bound is set for the diets that restrict methionine intake.

**Nutrient ID**	**Nutrient name**	**Minimum intake**	**Maximum intake**	**Minimum intake (% of calories)**	**Maximum intake (% of calories)**
203	Protein	$56^{\text{C, O, V, M, I}}$, 46^{II} [g]	$\text{ND}^{\text{C, O, V, M, I, II}}$	$10^{\text{C, O, V, M, I, II}}$	$35^{\text{C, O, V, M, I, II}}$
204	Total lipid	$0^{\text{C, O, V, M, I, II}}$ [g]	$\text{ND}^{\text{C, O, V, M, I, II}}$	$20^{\text{C, O, V, M, I, II}}$	$35^{\text{C, O, V, M, I, II}}$
205	Carbohydrate	$130^{\text{C, O, V, M, I, II}}$ [g]	$\text{ND}^{\text{C, O, V, M, I, II}}$	$45^{\text{C, O, V, M, I, II}}$	$65^{\text{C, O, V, M, I, II}}$
291	Fiber	$38^{\text{C, O, V, M}}$, 30^{I}, 21^{II} [g]	$\text{ND}^{\text{C, O, V, M, I, II}}$	$\text{ND}^{\text{C, O, V, M, I, II}}$	$\text{ND}^{\text{C, O, V, M, I, II}}$
301	Calcium	$1000^{\text{C, O, V, M}}$, $1200^{\text{I, II}}$ [mg]	$2500^{\text{C, O, V, M, I, II}}$ [mg]	$\text{ND}^{\text{C, O, V, M, I, II}}$	$\text{ND}^{\text{C, O, V, M, I, II}}$
303	Iron	$8^{\text{C, O, V, M, I, II}}$ [mg]	$45^{\text{C, O, V, M, I, II}}$ [mg]	$\text{ND}^{\text{C, O, V, M, I, II}}$	$\text{ND}^{\text{C, O, V, M, I, II}}$
304	Magnesium	$400^{\text{C, O, V, M}}$, 420^{I}, 320^{II} [mg]	$\text{ND}^{\text{C, O, V, M, I, II}}$	$\text{ND}^{\text{C, O, V, M, I, II}}$	$\text{ND}^{\text{C, O, V, M, I, II}}$
305	Phosphorus	$700^{\text{C, O, V, M, I, II}}$ [mg]	$4000^{\text{C, O, V, M, I, II}}$ [mg]	$\text{ND}^{\text{C, O, V, M, I, II}}$	$\text{ND}^{\text{C, O, V, M, I, II}}$
306	Potassium	$4700^{\text{C, O, V, M, I, II}}$ [mg]	$\text{ND}^{\text{C, O, V, M, I, II}}$	$\text{ND}^{\text{C, O, V, M, I, II}}$	$\text{ND}^{\text{C, O, V, M, I, II}}$
309	Zinc	$11^{\text{C, O, V, M, I}}$, 8^{II} [mg]	$40^{\text{C, O, V, M, I, II}}$ [mg]	$\text{ND}^{\text{C, O, V, M, I, II}}$	$\text{ND}^{\text{C, O, V, M, I, II}}$
312	Copper	$0.9^{\text{C, O, V, M, I, II}}$ [mg]	$10^{\text{C, O, V, M, I, II}}$ [mg]	$\text{ND}^{\text{C, O, V, M, I, II}}$	$\text{ND}^{\text{C, O, V, M, I, II}}$
315	Manganese	$2.3^{\text{C, O, V, M, I}}$, 1.8^{II} [mg]	$11^{\text{C, O, V, M, I, II}}$ [mg]	$\text{ND}^{\text{C, O, V, M, I, II}}$	$\text{ND}^{\text{C, O, V, M, I, II}}$
317	Selenium	$55^{\text{C, O, V, M, I, II}}$ [µg]	$400^{\text{C, O, V, M}}$, $402^{\text{I, II}}$ [µg]	$\text{ND}^{\text{C, O, V, M, I, II}}$	$\text{ND}^{\text{C, O, V, M, I, II}}$
320	Vitamin A	$900^{\text{C, O, V, M, I}}$, 700^{II} [µg RAE]	$3000^{\text{C, O, V, M, I, II}}$ [µg RAE]	$\text{ND}^{\text{C, O, V, M, I, II}}$	$\text{ND}^{\text{C, O, V, M, I, II}}$
323	Vitamin E	$15^{\text{C, O, V, M, I, II}}$ [mg]	$1000^{\text{C, O, V, M, I, II}}$ [mg]	$\text{ND}^{\text{C, O, V, M, I, II}}$	$\text{ND}^{\text{C, O, V, M, I, II}}$
328	Vitamin D	$5^{\text{C, O, V, M}}$, $10^{\text{I, II}}$ [µg]	$50^{\text{C, O, V, M, I, II}}$ [µg]	$\text{ND}^{\text{C, O, V, M, I, II}}$	$\text{ND}^{\text{C, O, V, M, I, II}}$
401	Vitamin C	$90^{\text{C, O, V, M, I}}$, 75^{II} [mg]	$2000^{\text{C, O, V, M, I, II}}$ [mg]	$\text{ND}^{\text{C, O, V, M, I, II}}$	$\text{ND}^{\text{C, O, V, M, I, II}}$
404	Thiamin	$1.2^{\text{C, O, V, M, I}}$, 1.1^{II} [mg]	$\text{ND}^{\text{C, O, V, M, I, II}}$	$\text{ND}^{\text{C, O, V, M, I, II}}$	$\text{ND}^{\text{C, O, V, M, I, II}}$
405	Riboflavin	$1.3^{\text{C, O, V, M, I}}$, 1.1^{II} [mg]	$\text{ND}^{\text{C, O, V, M, I, II}}$	$\text{ND}^{\text{C, O, V, M, I, II}}$	$\text{ND}^{\text{C, O, V, M, I, II}}$
406	Niacin	$16^{\text{C, O, V, M, I}}$, 14^{II} [mg NE]	$35^{\text{C, O, V, M, I, II}}$ [mg]	$\text{ND}^{\text{C, O, V, M, I, II}}$	$\text{ND}^{\text{C, O, V, M, I, II}}$

407	Sodium	ND[C, O, V, M, I, II]	2300[C, O, V, M, I, II]	ND[C, O, V, M, I, II]	ND[C, O, V, M, I, II]
410	Pantothenic acid	5[C, O, V, M, I, II] [mg]	ND[C, O, V, M, I, II]	ND[C, O, V, M, I, II]	ND[C, O, V, M, I, II]
415	Vitamin B_6	1.3[C, O, V, M], 1.7[I], 1.5[II] [mg]	100[C, O, V, M, I, II] [mg]	ND[C, O, V, M, I, II]	ND[C, O, V, M, I, II]
418	Vitamin B_{12}	2.4[C, O, M, I, II], NA[V] [µg]	ND[C, O, V, M, I, II]	ND[C, O, V, M, I, II]	ND[C, O, V, M, I, II]
421	Choline	550[C, O, V, M, I], 425[II] [mg]	3500[C, O, V, M], 5500[I, II] [mg]	ND[C, O, V, M, I, II]	ND[C, O, V, M, I, II]
430	Vitamin K	120[C, O, V, M, I], 90[II] [µg]	ND[C, O, V, M, I, II]	ND[C, O, V, M, I, II]	ND[C, O, V, M, I, II]
435	Folate	400[C, O, V, M, I, II] [µg DFE]	1000[C, O, V, M, I, II] [µg DFE]	ND[C, O, V, M, I, II]	ND[C, O, V, M, I, II]
501	Tryptophan	0.005[C, O, V, M, I, II] [g/kg]	ND[C, O, V, M, I, II]	ND[C, O, V, M, I, II]	ND[C, O, V, M, I, II]
502	Threonine	0.02[C, O, V, M, I, II] [g/kg]	ND[C, O, V, M, I, II]	ND[C, O, V, M, I, II]	ND[C, O, V, M, I, II]
503	Isoleucine	0.019[C, O, V, M, I, II] [g/kg]	ND[C, O, V, M, I, II]	ND[C, O, V, M, I, II]	ND[C, O, V, M, I, II]
504	Leucine	0.042[C, O, V, M, I, II] [g/kg]	ND[C, O, V, M, I, II]	ND[C, O, V, M, I, II]	ND[C, O, V, M, I, II]
505	Lysine	0.038[C, O, V, M, I, II] [g/kg]	ND[C, O, V, M, I, II]	ND[C, O, V, M, I, II]	ND[C, O, V, M, I, II]
506	Methionine	0.019[C, O, V, M, I, II] [g/kg]	ND[C, O, V], 0.0209[M, I, II] [g/kg]*	ND[C, O, V, M, I, II]	ND[C, O, V, M, I, II]
508	Phenylalanine	0.033[C, O, V, M, I, II] [g/kg]	ND[C, O, V, M, I, II]	ND[C, O, V, M, I, II]	ND[C, O, V, M, I, II]
510	Valine	0.024[C, O, V, M, I, II] [g/kg]	ND[C, O, V, M, I, II]	ND[C, O, V, M, I, II]	ND[C, O, V, M, I, II]
512	Histidine	0.014[C, O, V, M, I, II] [g/kg]	ND[C, O, V, M, I, II]	ND[C, O, V, M, I, II]	ND[C, O, V, M, I, II]
601	Cholesterol	ND[C, O, V, M, I, II]	300[C, O, V, M, I, II] [mg]	ND[C, O, V, M, I, II]	ND[C, O, V, M, I, II]
605	Trans fat	ND[C, O, V, M, I, II]	ND[C, O, V, M, I, II]	0[C, O, V, M, I, II]	1[C, O, V, M, I, II]
606	Saturated fat	ND[C, O, V, M, I, II]	ND[C, O, V, M, I, II]	0[C, O, V, M, I, II]	10[C, O, V, M, I, II]
675	Linoleic acid	17[C, O, V, M], 14[I], 11[II] [g]	ND[C, O, V, M, I, II]	5[C, O, V, M, I, II]	10[C, O, V, M, I, II]
851	α-Linolenic acid	1.6[C, O, V, M, I], 1.1[II] [g]	ND[C, O, V, M, I, II]	0.6[C, O, V, M, I, II]	1.2[C, O, V, M, I, II]

Supplementary Table S2. Foods with high nutritional fitness (NF) across diets (extension of Tables 1 and 3). *x* (avg. ± s.d.) denotes the average and standard deviation of the weights of a food (g per day) in irreducible food sets containing that food. For each food in Tables 1 and 3, we present the diets that give NF > 0.7 [C, control diet; O, ovo-lacto vegetarian diet; V, vegan diet; M, methionine-restricted diet; **I**, personalised diet **I** (61-year-old male); **II**, personalised diet **II** (58-year-old female)], along with the specific values of NF and *x* in parentheses beside each diet.

(**a**) Extension of Table 1 for control, ovo-lacto vegetarian, vegan, and methionine-restricted diets.

**Food category**	**Food**	**Diet with high nutritional fitness**
Protein-rich	Whole milk	O (NF=0.86; *x*=1097±239)
	Nonfat dry milk, reduced fat milk, 1%-fat milk	O (NF=0.83; *x*=648±579)
	Snapper	C (NF=0.83; *x*=459±77)
	Ocean perch	C (NF=0.80; *x*=505±86)
	Roe	C (NF=0.70; *x*=65±19), M (NF=0.79; *x*=70±13)
Fat-rich	Almond	C (NF=0.97; *x*=179±27), O (NF=0.97; *x*=171±33), V (NF=0.97; *x*=167±30), M (NF=0.99; *x*=183±27)
	Chia seed	C (NF=0.87; *x*=17±6), O (NF=0.95; *x*=15±5), V (NF=0.98; *x*=18±6), M (NF=0.93; *x*=17±6)
	Dried pumpkin and squash seed kernels	C (NF=0.84; *x*=119±28), O (NF=0.87; *x*=114±27), V (NF=0.87; *x*=128±33)
	Pork separable fat	C (NF=0.80; *x*=136±8)
	Dried black walnut	O (NF=0.71; *x*=100±22), V (NF=0.77; *x*=102±18)
Carbohydrate-rich	Cherimoya	C (NF=0.96; *x*=1622±306), O (NF=0.91; *x*=1499±266), V (NF=0.72; *x*=1394±325), M (NF=0.89; *x*=1608±253)
	Frozen immature lima bean	O (NF=0.72; *x*=1177±445), V (NF=0.85; *x*=1487±421)
	Frozen green pea	O (NF=0.76; *x*=990±288), V (NF=0.80; *x*=1011±216)
	Tangerine	C (NF=0.76; *x*=2796±613), V (NF=0.78; *x*=2772±582), M (NF=0.77; *x*=2928±374)
	Full-fat soy flour	V (NF=0.76; *x*=160±49)
Low-macronutrient	Ultraviolet-treated portabella	O (NF=0.87; *x*=169±135), V (NF=0.94; *x*=137±97)
	Maitake	O (NF=0.88; *x*=110±69), V (NF=0.87; *x*=88±62)
	Dried shiitake	O (NF=0.87; *x*=122±15)
	Red cabbage	C (NF=0.74; *x*=1683±442), O (NF=0.71; *x*=1457±442), M (NF=0.83; *x*=2012±323)
	Chanterelle	O (NF=0.80; *x*=121±76), V (NF=0.76; *x*=110±44)

(**b**) Extension of Table 3 for personalised diet I (61-year-old male) and personalised diet II (58-year-old female).

**Food category**	**Food**	**Diet with high nutritional fitness**
Protein-rich	Dried smelt	I (NF=0.82; x=19±3), II (NF=0.90; x=14±1)
	Dried whitefish	I (NF=0.88; x=19±3), II (NF=0.87; x=14±1)
	Dried chum salmon	II (NF=0.74; x=12±3)
	Common octopus	II (NF=0.72; x=14±1)
Fat-rich	Almond	I (NF=0.95; x=139±23), II (NF=0.96; x=137±22)
	Chia seed	I (NF=0.94; x=14±4), II (NF=0.87; x=15±4)
	Dried pumpkin and squash seed kernels	I (NF=0.82; x=97±25), II (NF=0.84; x=96±23)
Carbohydrate-rich	Cherimoya	II (NF=0.80; x=1123±236)
	Frozen immature lima bean	I (NF=0.76; x=1188±216)
Low-macronutrient	Ultraviolet-treated portabella	I (NF=0.89; x=185±85), II (NF=0.95; x=203±104)
	Maitake	I (NF=0.88; x=98±55), II (NF=0.84; x=107±60)
	Zucchini	II (NF=0.87; x=2795±395)
	Hubbard squash	I (NF=0.81; x=3211±325)
	Dandelion green	I (NF=0.74; x=445±143), II (NF=0.77; x=466±154)
	Chanterelle	II (NF=0.73; x=224±64)

Supplementary Methods

Food and nutritional data and various diets

1. Diet and physical conditions

In the current study, we consider the following four diet styles of a physically active, 20-year-old male with standard height and weight: (*i*) control, (*ii*) ovo-lacto vegetarian, (*iii*) vegan, and (*iv*) methionine-restricted diets. In addition, we consider two hypothetical scenarios of highly personalised, methionine-restricted diets of (*v*) a 61-year-old male with low physical activity and (*vi*) a physically active 58-year-old female. The details of these diet and physical conditions are explained below.

2. Nutritional composition of food

For diets (*i*) and (*iv*), we use the data of food nutritional compositions in our previous study[1], wherein we accessed the USDA National Nutrient Database for Standard Reference, Release 24[2]. The database provides the contents of 7,907 foods in terms of their energy (calorie) and nutrients. The nutrient contents were normalised to the sum (100 g) of protein, total lipid, carbohydrate, water, ash, and alcohol of each food. From these foods, we considered raw foods, as well as other foods whose nutrient contents have been minimally modified. Specifically, we selected foods that fall into one of the following categories. First, we selected foods obtained directly from nature or directly from agriculture, fishery, or livestock farming, without any explicitly added or fortified ingredients such as salt, sugar, and vitamins. Those foods include various raw vegetables, fruits, meat, and fish. Second, we selected foods that belong to the first category but have some modifications in their physical properties. Those foods include ground products, e.g., wheat flour and ground meat. Third, we selected foods that belong to the first or second category but have additional, minor modifications in their nutrient contents, i.e., frozen, dried, low-fat, non-fat, and ultraviolet-treated products. In total, 1,068 foods were selected for diets (*i*) and (*iv*), and here, all of them are just called raw foods. For diet (*iii*), we only consider plant-derived foods among them, and for diet (*ii*), egg and milk products additionally. Diets (*v*) and (*vi*) include the plant-derived foods in diet (*iii*), along with limited amounts of eggs, whole milk, and certain types of fish (see below and Supplementary Data S2). Furthermore, diets (*v*) and (*vi*) include cheese products, although cheese products themselves do not belong to raw foods.

3. Consolidation of foods that have almost identical nutrient contents

In the previous study[1], we consolidated raw foods that have almost identical nutrient contents by calculating the following quantity for foods *i* and *j*:

$$F_{ij} = \min_{K \geq 0} \frac{1}{n_{ij}} \sum_{m=1}^{n_{ij}} \frac{\left(Ka_{im} - a_{jm}\right)^2}{\left(Ka_{im} + a_{jm}\right)^2},$$

where $\alpha_{i(j)m}$ is the density of nutrient *m* in food *i* (*j*), n_{ij} is the total number of nutrient *m*'s for foods *i* and *j*, and *K* is a positive real number selected to minimise F_{ij} given $\{\alpha_{im}\}$ and $\{\alpha_{jm}\}$. We only considered nutrient *m*'s that have explicit records of their quantities in both foods *i* and *j* and have non-zero quantities for food *i* or *j*. The resulting F_{ij} ranges from zero to one, and a small F_{ij} indicates that the foods *i* and *j* are similar in their relative nutrient amounts. The calculation of F_{ij} works even with nutrients on very different scales or with different units for the quantities (e.g., μg RAE for vitamin A, and μg DFE for folate). From a probability distribution of F_{ij} over all pairs of foods *i* and *j*, we found a sharp transition of the distribution at $F_{ij} \sim 0.012$. Accordingly, we created unified groups of foods; each group forms an isolated, single connected component in a network of foods linked through $F_{ij} < 0.012$. For each unified group, the nutrient quantities (per 100 g) were averaged over the foods. The averages for the nutrients were calculated only from foods that had explicit records for the nutrient quantity and were not dried or frozen (differences in the water contents of foods cause large variations

in nutrient densities, despite the similar nutrient compositions of the foods). By treating each unified group as a single food, we obtained a total of 653 raw foods (Supplementary Data S1; unlike the previous study[1], here we do not consider sea cucumber, because of its suspicious nutrient content data). In the present study, the aforementioned cheese products for diets (*v*) and (*vi*) were similarly consolidated into 16 cheese products by unifying the foods linked through $F_{ij} < 0.06$ (Supplementary Data S2).

4. Identification of food categories

We follow our previous grouping of foods[1] based on their nutritional similarity. We conducted an average-linkage hierarchical clustering of the foods (agglomerating foods by the large nutritional similarity, as described in the previous study[1]) and built a dendrogram, in which each leaf is a food and branches represent groups of foods. Groups that are deeper in the hierarchical levels from the root to leaves contain foods with greater nutritional similarity than less deep groups. Near the root, six foods (raw, dried, and frozen egg whites, duck and goose fat, honey, and table salt) are first split from the others because their nutrient contents are dissimilar to those of most foods. The remaining foods are divided into two large parts – animal-derived and plant-derived. The animal-derived part has a layered, core-peripheral organisation: the core region (bulky clusters of foods) at the deeper hierarchical level includes protein-rich foods, while the peripheral region outside the core includes both protein-rich and fat-rich foods. In a similar fashion, the plant-derived part is divided into protein-rich, fat-rich, carbohydrate-rich, and low-macronutrient categories (Supplementary Data S1; the 'low-calorie' category in our previous study is called here the 'low-macronutrient' category, in order to prevent any nomenclatural confusion later).

5. Recommended levels of nutrient intakes

For the recommended daily levels of nutrient intakes, as in our previous study[1], we referred to the Dietary Reference Intakes (DRI) published by the Institute of Medicine of the National Academies[3], the Dietary Guidelines for Americans 2010[4], and the 2002 Joint WHO/FAO Expert Consultation recommendations[5]. We mainly used the data from the first source, while the second and third sources were references only for the data on cholesterol, saturated fatty acids, and *trans*-fatty acids.

The specific values for the lower and upper bounds of the recommended daily intake of nutrients depend on ages and genders. For diets (*i*) to (*v*), the daily recommended energy E was calculated following the formula E (kcal, for a ≥19-year-old male) = $662 - (9.53 \times y) + P_a \times (15.91 \times w + 539.6 \times h)$, where y denotes the age in years, P_a stands for the physical activity level, w is the weight in kg, and h is the height in m. For diets (*i*) to (*iv*) and for diet (*v*), $y = 20$ and 61, $P_a = 1.25$ and 1.11, $w = 70$ and 73, and $h = 1.77$ and 1.68, respectively. For diet (*vi*), we use the formula E (kcal, for a ≥19-year-old non-pregnant female) = $354 - (6.91 \times y) + P_a \times (9.36 \times w + 726 \times h)$ with $y = 58$, $P_a = 1.25$, $w = 62$, and $h = 1.70$.

Unlike our previous study[1], here we do not impose any lower bound of sodium intake (Supplementary Table S1), under the assumption that the recommended sodium intake is readily achievable in common diets through the consumption of added salt, not necessarily only through raw food consumption. In the case of diet (*iii*), we do not impose any lower bound of vitamin B_{12} intake (Supplementary Table S1). This is because a linear programming (LP) problem with variable food weights to satisfy the recommended daily nutrient intake gives an infeasible solution, as long as the lower bound of the recommended vitamin B_{12} intake is exerted in the diet (*iii*) (see below for the details). For diets (*iv*) to (*vi*), we impose the very tight upper bound of methionine intake, as merely 10% more than the lower bound of the methionine intake (Supplementary Table S1).

Nutritional fitness of foods across diets

To calculate the nutritional fitness (NF) of each food, we start by constructing irreducible food sets; each of which is a set of a small number of different foods[1]. These foods satisfy our daily nutrient demands, and they are not a superset of any other irreducible food set. To obtain a collection of irreducible food sets, we generated an initial food set by solving the following mixed-integer linear programming (MILP) problem:

$$\text{Minimise} \sum_i q_i$$

Subject to:

$$0 \le x_i \le q_i W$$

$$\sum_i e_i x_i = E$$

$$L_j \le \sum_i a_{ij} x_i \le U_j$$

$$Q_j E \le \sum_i a_{ij} c_{ij} x_i \le R_j E$$

$$\sum_i x_i \le W$$

where q_i is a binary variable (if food i is in the food set, $q_i = 1$; otherwise, $q_i = 0$), x_i is a real variable for the weight of food i to consume per day, E is the daily recommended energy (calorie) that we described above, L_j (U_j) is the lower (upper) bound of the daily recommended intake of nutrient j (Supplementary Table S1), Q_j (R_j) is similar to L_j (U_j) but defined by the % of total energy (Supplementary Table S1), W is the limit of the total weight of daily food consumption (W = 4 kg in this study), e_i is the energy density of food i, α_{ij} is the density of nutrient j in food i, and c_{ij} is the energy density of nutrient j in food i. In the case of diet (*iii*), it was infeasible to find the solution to the above MILP problem as long as L_j of vitamin B_{12} is exerted. It was even infeasible to find the solution to the corresponding LP problem with q_i = 1 for every food i in diet (*iii*). Therefore, we set L_j of vitamin B_{12} to zero in the case of diet (*iii*). For methionine in diets (*iv*) to (*vi*), we set $U_j = 1.1\ L_j$ to restrict methionine intake. In the cases of highly personalised diets (*v*) and (*vi*), we add the constraint $\sum_{i \in S} x_i \le D_S$ where S is the collection of certain foods and D_S is the limit of the daily consumed amount of foods in S. Specifically, S corresponds to the following foods in Supplementary Data S2: eggs [D_S = 12.86 g and 6.43 g for diets (*v*) and (*vi*), respectively, which mean ~2 eggs/week and ~1 egg/week], whole milk [D_S = 22.29 g and 7.43 g for diets (*v*) and (*vi*), respectively, which mean ~150 ml/week and ~50 ml/week], fish [D_S = 21.43 g and 14.29 g for diets (*v*) and (*vi*), respectively, which mean 150 g/week and 100 g/week], and cheese [D_S = 5.71 g and 2.86 g for diets (*v*) and (*vi*), respectively, which mean 40 g/week and 20 g/week].

The solution to this MILP problem in each diet gave a food set with the minimum size (i.e., minimum $\sum_i q_i$). Next, we expanded the collection of food sets by subsequently adding new food sets to the collection. At each step of adding a new food set, this food set is a solution of the above MILP problem, and is constrained to not be a superset of any previous food set in the collection. We only considered food sets with $\sum_i q_i$ < 6, 7, 7, 6, 7, and 7 for diets (*i*) to (*vi*), respectively. If it was not feasible to find more food sets for the collection, the process was terminated. The final collection comprises 52,957, 43,924, 20,713, 4,101, 1,053, and 5,225 irreducible food sets in total for diets (*i*), (*ii*), (*iii*), (*iv*), (*v*), and (*vi*), respectively. Mathematically, such a collection of irreducible food sets is uniquely determined and has no degeneracy. For every diet except diet (*i*), irreducible food sets were first obtained using IBM ILOG CPLEX solver (v. 12.4) and subsequently obtained using Gurobi solver

(v. 7.0.2); for diet (*i*), we only applied IBM ILOG CPLEX solver, to reduce an otherwise excessively long computation time.

The NF_i of food *i* is given by $NF_i = \log(f_i+1)/\log(N+1)$, where f_i is the number of irreducible food sets including food *i*, and *N* is the total number of irreducible food sets. NF_i ranges from zero to one, and a large NF_i indicates that food *i* is nutritionally favourable. For the generalised definition of NF_i, any functional form that monotonically increases with f_i is acceptable, as long as only ordinal information of NF_i matters. Note that f_i is capable of quantifying NF_i under the condition of small $\sum_i q_i$ as in this study. Otherwise, it may be hard to estimate the true nutritional adequacy of food *i'* using solely $f_{i'}$. For example, a nutritionally poor food *i'* in an irreducible food set will be easily complemented by many other foods (in the same set) to satisfy the above constraints if $\sum_i q_i$ is not small enough.

Key nutrients relevant to each food or diet

1. Key nutrients contributing to the NF of each food

To identify the individual nutrients responsible for the NFs of foods, we measure the following quantity ϕ_{ij} for each pair of food *i* and nutrient *j*:

$$\phi_{ij} = \left\langle \frac{a_{ij}x_i}{\sum_k a_{kj}x_k} \right\rangle,$$

where α_{ij} is the density of nutrient *j* in food *i*, x_i is the weight of food *i* to consume per day in a given irreducible food set, and $\langle\cdot\rangle$ is an average over all irreducible food sets that include the food *i* (if $\sum_k \alpha_{kj}x_k = 0$ in any irreducible food set, this irreducible food set is excluded from the calculation). In other words, ϕ_{ij} represents the food *i*'s contribution to the total amount of the nutrient *j* in an irreducible food set, on average. The value of ϕ_{ij} ranges from zero to one (Supplementary Data S1 and S2). We interpret the nutrient *j* with large ϕ_{ij} as the main contributor to the food *i*'s NF. For a given value ϕ_{ij}, we tested its statistical significance by calculating the one-sided *P* value of how frequently $\phi_{i'j}$ of a randomly-chosen raw food *i'* is greater than or equal to ϕ_{ij} (if the raw food *i'* did not appear in any irreducible food sets, $\phi_{i'j}$ was treated as zero in this calculation).

Because of the possible presence of $x_{i(k)}$'s multiple solutions within each irreducible food set resulting from the aforementioned MILP problem, the specific ϕ_{ij} value may vary depending on those multiple solutions. To address this multiple-solution issue, we maximised or minimised each x_i in a given irreducible food set and thereby found the x_i's range allowed by the multiple solutions, while maintaining all the previous constraints of the MILP problem for this irreducible food set. A relative difference between the maximum (or minimum) and original x_i values [i.e., $|x_i^{\max(\min)} - x_i^{\mathrm{org}}| / x_i^{\mathrm{org}}$ with $x_i^{\max(\min)}$ and x_i^{org} for the maximum (minimum) and original x_i values, respectively] is found to be less than ~0.2 to ~0.35 for the majority (70%) of x_i values in every diet. Given this limited variation of x_i, the central limit theorem is expected to be applied for the calculation of ϕ_{ij}, which involves a rough 'average' of x_i over the irreducible food sets having the food *i* (see the above definition of ϕ_{ij}). Therefore, the variation of ϕ_{ij} from the multiple solutions is unlikely to be large if food *i* has high NF and thus belongs to many irreducible food sets.

2. Nutrients at risk of deficiency in each diet

For each diet, we examined whether a given nutrient *j* is subjected to a risk of deficiency in its daily intake, through the calculation of the following quantity θ_j:

$$\theta_j = \left\langle \frac{\max\left(\sum_k a_{kj} x_k\right) - L_j}{U_j - L_j} \right\rangle, \text{ or } \theta_j = \left\langle \frac{\max\left(\sum_k a_{kj} x_k\right) - L_j}{\max\left(\sum_k a_{kj} x_k\right)} \right\rangle,$$

where L_j (U_j) is the lower (upper) bound of the recommended daily intake of nutrient j, max(·) is the maximum value among all multiple solutions with altered x_k's in a given irreducible food set, and ⟨·⟩ is an average over all irreducible food sets. When calculating max(·), we maximised the value from a corresponding irreducible food set, while maintaining all the previous constraints of the MILP problem for this irreducible food set. If the nutrient j has the upper bound of its recommended daily intake, we calculate the former θ_j, and otherwise, the latter θ_j. In other words, θ_j quantifies the nutrient j's maximally possible excess over its minimally required intake level in an irreducible food set, on average. The value of θ_j ranges from zero to one, and small θ_j value indicates a risk of nutrient j's deficiency in a given diet.

For the nutrients having the recommended daily intake of their calorie, α_{kj}, L_j, and U_j in θ_j are substituted for by $\alpha_{kj}c_{kj}$, Q_jE, and R_jE, respectively, where c_{kj} is the energy density of nutrient j in food k, E is the daily recommended energy (calorie), and Q_j (R_j) is similar to L_j (U_j) but defined by the % of total energy. For the nutrients that have both L_j (or U_j) and Q_j (or R_j), we calculate both θ_j's, and take a smaller value (i.e., a value with a stricter condition) between the two θ_j's.

Correlation between selenium and protein levels across foods

1. Calculation of the Pearson correlation

We calculated the Pearson correlation between the densities of selenium and protein across raw foods[1]. Each selenium or protein density was measured as the quantity per dry weight. Only raw foods having explicit records of both selenium and protein amounts (and at least one of them with a non-zero quantity) were considered.

Note that the Pearson correlation coefficient (r) between two variables X_i and Y_i (i = 1, 2,⋯, N) is easily distorted by the presence of outliers. When we measured the Pearson correlation, we excluded outliers as follows: $x_i = (X_i - \mu_x)/\sigma_x$ and $y_i = (Y_i - \mu_y)/\sigma_y$, where $\mu_{x(y)}$ and $\sigma_{x(y)}$ are the average and standard deviation of X_i (Y_i), respectively. In a Cartesian plane, we drew a link connecting the data points $P_i = (x_i, y_i)$ and $P_{i'} = (x_{i'}, y_{i'})$ if the Euclidean distance between P_i and $P_{i'}$ was shorter than a certain cut-off d_c (we chose $d_c = \sqrt{3}$). In this 'network' of data points, we identified the data points in the largest connected component and considered the others to be outliers. The Pearson correlation was measured only for the data points in the largest connected component.

2. Statistical significance

The statistical significance of the correlation (r) between selenium and protein densities across raw foods was tested as follows[1]: we first remove the outlier raw foods defined above before generating the null model. Next, we select only the raw foods having explicit records for both selenium and protein amounts (and at least one among them with a non-zero quantity), and we randomly shuffle the densities (quantity per dry weight) of either selenium or protein across those raw foods. The Pearson correlations between such selenium and protein densities across the raw foods constitute the null distribution that gives a P value.

We measured a P value as follows. Let $\{\alpha_i\}$ (i = 1, 2,⋯, N) be a sequence of random numbers in ascending order from a null distribution. Using $\{\alpha_i\}$, we obtain the two-sided P value of a given number X (= r) as follows: a value Λ (P =1 if X = Λ) is expressed as

$$\Lambda = \begin{cases} a_{k+1}, & \text{if } N = 2k+1 \text{ for an integer } k \\ \frac{a_k + a_{k+1}}{2}, & \text{if } N = 2k \text{ for an integer } k \end{cases},$$

and the two-sided P value of X is given by

$$P = \begin{cases} \left(\frac{\#\text{ of } a_i \text{ satisfying } a_i \leq X}{\#\text{ of } a_i \text{ satisfying } a_i \leq \Lambda}\right), & \text{if } X \leq \Lambda \\ \left(\frac{\#\text{ of } a_i \text{ satisfying } a_i \geq X}{\#\text{ of } a_i \text{ satisfying } a_i \geq \Lambda}\right), & \text{if } X > \Lambda \end{cases}.$$

If $P < 2 \times 10^{-3}$ for a given value of X (= r), we extrapolate the P value using the estimation $P \propto (1 - |X|)^{\gamma}$ at $X \to \pm 1$ (i.e., at $r \to \pm 1$; γ depends on the sign of X, and is estimated from the null distribution of X).

References

1. Kim, S., Sung, J., Foo, M., Jin, Y.-S. & Kim, P.-J. Uncovering the nutritional landscape of food. *PLoS One* **10,** e0118697 (2015).
2. U.S. Department of Agriculture, Agricultural Research Service. *USDA National Nutrient Database for Standard Reference, Release 24. Nutrient Data Laboratory Online.* http://www.ars.usda.gov/Services/docs.htm?docid=22808 (Accessed: 31st December 2011).
3. National Research Council. *Dietary Reference Intakes for Energy, Carbohydrate, Fiber, Fat, Fatty Acids, Cholesterol, Protein, and Amino Acids (Macronutrients)* (National Academies Press, 2005).
4. U.S. Department of Agriculture & U.S. Department of Health and Human Services. *Dietary Guidelines for Americans, 2010* (U.S. Government Printing Office, 2010).
5. Nishida, C., Uauy, R., Kumanyika, S. & Shetty, P. The joint WHO/FAO expert consultation on diet, nutrition and the prevention of chronic diseases: process, product and policy implications. *Public Health Nutr.* **7,** 245–250 (2004).
